# High Sensitivity and NPV for BinaxNOW Rapid Antigen Test in Children at a Mass Testing Site during Prevalent Delta Variant Period

**DOI:** 10.1128/spectrum.00236-22

**Published:** 2022-06-28

**Authors:** Kristie J. Sun, Mary Jane E. Vaeth, Matthew Robinson, Maryam Elhabashy, Ishaan Gupta, Sophia Purekal, E. Adrianne Hammershaimb, Ria Peralta, Asia Mitchell, Maisha Foyez, J. Kristie Johnson, James R. Ficke, Yukari C. Manabe, James D. Campbell, Charles W. Callahan, Charles F. Locke, Melinda Kantsiper, CONQUER COVID Consortium, Zishan K. Siddiqui

**Affiliations:** a Case Western Reserve University School of Medicine, Cleveland, Ohio, USA; b Baltimore Convention Center Field Hospital, Baltimore, Maryland, USA; c Department of Medicine, The Johns Hopkins University School of Medicine, Baltimore, Maryland, USA; d University of Maryland Baltimore County, Baltimore, Maryland, USA; e Center for Vaccine Development and Global Health, Department of Pediatrics, University of Maryland School of Medicine, Baltimore, Maryland, USA; f Nova Southeastern University College of Osteopathic Medicine, Fort Lauderdale, Florida, USA; g Department of Pathology, University of Maryland School of Medicine, Baltimore, Maryland, USA; h Department of Orthopedic Surgery, The Johns Hopkins University, Baltimore, Maryland, USA; i Division of Hospital Medicine, The Johns Hopkins Bayview Medical Center, Baltimore, Maryland, USA; Keck School of Medicine of the University of Southern California; Division of Internal Medicine, University of Maryland School of Medicine, Baltimore, Maryland, USA; Division of Hospital Medicine, Johns Hopkins Bayview Medical Center, Baltimore, Maryland, USA; Division of Hospital Medicine, Johns Hopkins Bayview Medical Center, Baltimore, Maryland, USA; Division of Internal Medicine, University of Maryland School of Medicine, Baltimore, Maryland, USA; Johns Hopkins Community Physicians, Baltimore, Maryland, USA

**Keywords:** COVID-19, pediatric rapid antigen testing

## Abstract

SARS-CoV-2 continues to develop new, increasingly infectious variants including delta and omicron. We evaluated the efficacy of the Abbott BinaxNOW Rapid Antigen Test against Reverse Transcription PCR (RT-PCR) in 1,054 pediatric participants presenting to a high-volume Coronavirus Disease 2019 (COVID-19) testing site while the delta variant was predominant. Both tests utilized anterior nares swabs. Participants were grouped by COVID-19 exposure and symptom status. 5.2% of samples tested positive by RT-PCR for SARS-CoV-2. For all participants, sensitivity of the BinaxNOW was 92.7% (95% CI 82.4%–98.0%), and specificity was 98.0% (95% CI 97.0%–98.8%). For symptomatic participants, positive predictive value (PPV) was 72.7% (95% CI 54.5%–86.7%) and negative predictive value (NPV) was 99.2% (95% CI 98.2%–100%). Among asymptomatic participants, PPV was 71.4% (95% CI 53.7%–85.4%) and NPV was 99.7% (95% CI 99.0%–100%). Our reported sensitivity and NPV are higher than other pediatric studies, potentially because of higher viral load from the delta variant, but specificity and PPV are lower.

**IMPORTANCE** The BinaxNOW rapid antigen COVID-19 test had a sensitivity of nearly 92% in both symptomatic and asymptomatic children when performed at a high-throughput setting during the more transmissible delta variant dominant period. The test may play an invaluable role in asymptomatic screening and keeping children safe in school.

## INTRODUCTION

Many U.S. children remain susceptible to SARS-CoV-2 infection. Twenty million children under 5 have no option for vaccination (1). Among the 28 million eligible children aged 5–11, 18% have received at least one dose of the Coronavirus Disease 2019 (COVID-19) vaccine as of December 8, 2021 (2). Similarly, only 51% of 25 million eligible children aged 12–17 years have received two doses of the vaccine (2). Quick, accurate, and accessible diagnostic testing for SARS-CoV-2 in pediatric populations is critical to keeping children in classrooms, especially given the transmissibility of newer variants (e.g., delta, omicron) among vaccinated individuals (3, 4). The impact of disrupted in-person learning has been substantial. Students have fallen behind in foundational coursework, with the effect compounded for students with historical racial or socioeconomic disadvantages (5).

One strategy to keep children safely in school is to incorporate routine testing. Challenges around testing include delayed result reporting, high cost for reverse transcription PCR (RT-PCR) tests, and access disparities among marginalized populations (6). Rapid antigen tests offer an attractive alternative, with results often returned in 15 min, lower cost, and ability to predict patients harboring culturable, infectious virus (7). Studies of the accuracy of these tests in children are conflicting. Most were conducted before the more transmissible and pathogenic variants like delta became prevalent (8, 9). We evaluated the real-world characteristics of the Abbott BinaxNOW rapid antigen test (Abbott Laboratories, Abbott Park, IL) in children presenting to a high-throughput setting. Our study took place in the context of a high prevalence of the delta variant, between May 7, 2021 and December 6, 2021. The proportion of COVID-19 cases due to the delta variant rapidly increased from less than 2.5% of cases to more than 99% in Maryland and surrounding states during the first month of the study period (10). The 7-day test positivity rate in Maryland at the start of the study was 3.44%, decreased to 0.54% by the end of June, and increased to 5.62% by the end of the study period (11).

## RESULTS

During the testing period, 1,054 of 2,811 children who were seen for diagnostic testing participated in the study. Among participants, 508 (48.3%) were female, 438 (41.6%) were White, 373 (35.4%) were African American, 105 (9.9%) were Hispanic, and the mean age was 8.9 years. Nonparticipants were similar for gender (female 49.1%, *P* = 0.6), were older (mean 9.4 years, *P* = 0.001), and were more often African American (55%, *P* < 0.0001) or Hispanic (15.1%, *P* < 0.0001). Symptomatic status was obtained for 1,046 (99.2%) participants, of which 756 (72.4%) were asymptomatic. Symptomatic participants were younger (7.8 vs 9.3 years, *P* < 0.0001) (Table 1). High-risk exposure was reported by 152 (20.1%) asymptomatic and 50 (17.2%) symptomatic participants. The COVID-19 prevalence rate, based on RT-PCR results, was 5.2% (55/1,054) overall, 9.0% (26/290) for symptomatic individuals, and 3.6% (27/756) for asymptomatic individuals. For symptomatic participants, 96.2% (275/286) tested within 7 days after symptom onset. The prevalence rate was 20% for symptomatic participants with high-risk exposure and 7.2% for asymptomatic with high-risk exposure (Table 2).

**TABLE 1:** Demographics of children presenting for COVID-19 diagnostic testing with concurrent RT-PCR and rapid antigen test, Baltimore, 2021

Characteristics	Asymptomatic	Symptomatic
	(N = 756)	(N = 290)
Female	49.9%	44.2%
Age (years)	9 (6–12)^*a*^	7 (4–11)^*a*^
Race		
White	40.0%	45.9%
African American	36.6%	32.5%
Other	23.4%	23.6%
Ethnicity		
Hispanic	10.1%	8.9%

a Data presented as median (interquartile range).

**TABLE 2:** Antigen test accuracy rates compared to RT-PCR as reference standard^*a*^

Exposure group	Positive/total (%)			
	RT-PCR	Rapid Antigen Test^*b*^	Sensitivity N (%) [95% CI]	Specificity N (%) [95% CI]
Overall	55/1054 (5.2)	71/1054 (6.7)	51/55 (92.7) [82.4–98.0]	979/999 (98.0) [96.9–98.8]
Symptomatic^*c*^ – all	26/290 (9.0)	33/290 (11.4)	24/26 (92.3) [74.9–99.1]	255/264 (96.6) [93.6–98.4]
Symptomatic – ≤7 days of symptoms	25/275 (9.1)	32/275 (11.6)	23/25 (92.0) [74.0–99.0]	241/250 (96.4) [93.3–98.3]
Symptomatic – high risk exposure	10/50 (20)	10/50 (20)	8/10 (80.0) [44.4–97.5]	38/40 (95.0) [83.1–99.4]
Asymptomatic^*c*^ – all	27/756 (3.6)	35/756 (4.6)	25/27 (92.6) [75.7–99.1]	719/729 (98.6) [97.5–99.3]
Asymptomatic – high risk exposure	11/152 (7.2)	10/152 (6.6)	9/11 (81.8) [48.2–97.7]	140/141 (99.3) [96.1–100.0]

a RT-PCR, reverse transcription PCR; CI, confidence interval.

b There were no inconclusive or invalid rapid antigen results.

c Symptom status data was missing for 8 participants, including 4 participants with rapid antigen positive results.

### Test accuracy.

**FIG 1:**
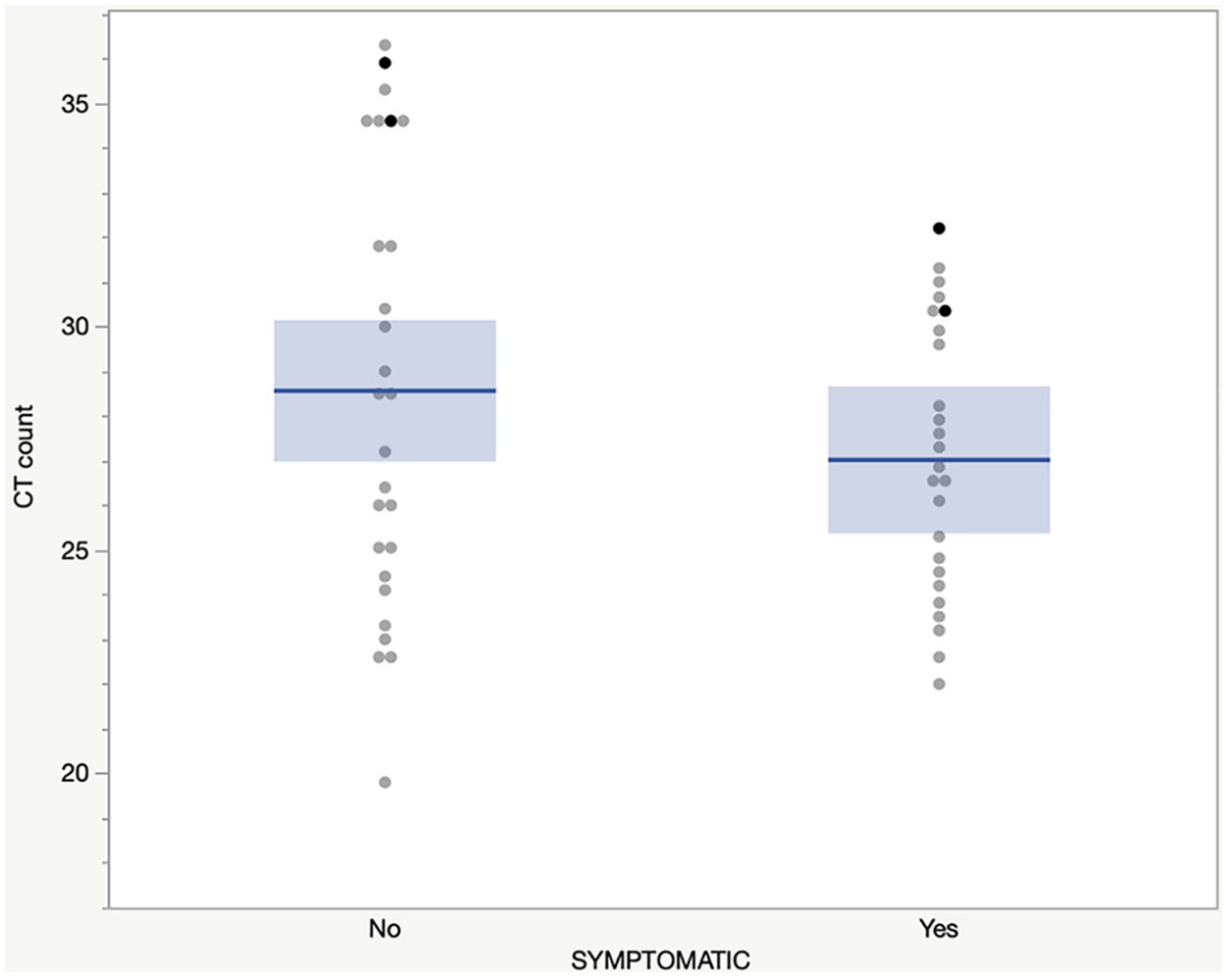
CT counts for symptomatic and asymptomatic participants. Darker dots represent false negative rapid antigen results.

The sensitivity of the rapid antigen tests for all participants was 92.7% (95% CI 82.4%–98.0%) and specificity was 98.0% (95% CI 97.0%–98.8%). Sensitivity was similar for symptomatic participants and asymptomatic participants (92.3% versus 92.6%) (*P* = 1.0). It was also similar for high-risk exposure groups, both symptomatic (80.0%; 95% CI 44.4%–97.5%) and asymptomatic (81.8%; 95% CI 48.2%–97.7%) (Table 2). The sensitivity for symptomatic patients tested within 7 or fewer days since onset of symptoms was 92.0% (95% CI 74.0–99.0). The specificity for symptomatic participants was 96.6% (95% CI 93.6%–98.4%) and for asymptomatic participants was 98.6% (95% CI 97.5%–99.3%). Among symptomatic individuals the positive predictive value (PPV) was 72.7% (95% CI 54.5%–86.7%) and the negative predictive value (NPV) was 99.2% (95% CI 98.2%–100%). Among asymptomatic individuals, the PPV was 71.4% (95% CI 53.7%–85.4%) and the NPV was 99.7% (95% CI 99.0%–100%). Mean C_T_ values were similar for the asymptomatic and symptomatic groups (28.6 versus 27; *P* = 0.2). The rapid antigen tests showed 100% sensitivity at C_T_ count 30 or below in both the symptomatic and asymptomatic populations (Fig. 1).

## DISCUSSION

This single center prospective study at a state-owned walk-up testing site showed high sensitivity, specificity, and NPV for the rapid antigen test in both symptomatic and asymptomatic children. The sensitivity was 92.3% in symptomatic participants, and all but one of these 26 RT-PCR positive participants were tested within 7 days of symptom onset. The sensitivity in the asymptomatic population was nearly identical at 92.6%, with an NPV of nearly 100%. Cycle threshold counts were similar for symptomatic and asymptomatic individuals, and rapid tests showed 100% sensitivity in both these groups at C_T_ count less than 30, which signifies higher viral loads and greater transmissibility. Our point estimate of BinaxNOW rapid antigen test sensitivity in children is above the 80% threshold set by the U.S. Food and Drug Administration (FDA) for emergency use authorization (EUA) for both symptomatic and asymptomatic children (12). Our results show that rapid antigen tests provide a reliable means to diagnose and screen for COVID-19 in children. Our findings of NPVs of 99.2% or greater for both symptomatic and asymptomatic children, including some children with recent high-risk exposures, should provide providers and parents with assurance that a negative rapid antigen test with this product can be trusted. These NPV values would be lower if prevalence increases above the 5% identified in our study. These data also provide support the use of these tests as part of a “test-to-stay” approach in schools (13). Because our study showed that roughly 30% of positive antigen tests were false positives, it would be prudent to confirm all positive antigen tests with a PCR test. While this does present an additional burden for students who test positive using rapid antigen tests, it would apply to a limited number of students, and far fewer than testing everyone with RT-PCR.

Our study shows higher sensitivity and NPV (8, 9). Differences could be related to higher viral load from prevalent delta variant during the study period as most of the prior pediatric studies were conducted before the delta variant became widespread (14). Additionally, ambient conditions for kit storage and use, variation in quality of the test kit between lots, and the skill of our experienced testers may also explain higher sensitivity in our study (15). Prior studies have reported a false positive rate of 0.5–9% (8, 16, 17). Our false positive rate of 2% is similar. Our study was conducted during a period of reduced social distancing and masking mandates compared with prior studies (11, 18). Higher prevailing respiratory virus infections may contribute to higher false positive rates since these may cross-react (19).

Our study has a few limitations. The results from a single site with over one year of experience in high volume testing may not be generalizable to all situations. However, with implementation of best practices, similar accuracy would be expected (20). Our study enrolled only a small number of children with high-risk exposure, and the estimates of accuracy had broad confidence intervals. However, the results are similar to overall symptomatic and asymptomatic groups. Additionally, three of the four false negative tests in the high-risk exposure group were tested within 3 days of exposure, and samples were likely collected too early for detection by a rapid antigen test. Finally, our control RT-PCR panel targeted only the N gene, which may be subject to mutation in future variants. BinaxNOW rapid antigen detects the N protein. This may also be a concern with variants with N-gene mutation. However, the RT-PCR used for the study detected 2 regions of the N gene, and mutation in both may not be as likely. RT-PCR tests that amplify multiple genes may be more robust in detecting future mutated strains. However, there were no concerns regarding the ability to detect prevalent variants with the genes amplified for our study. BinaxNOW rapid antigen tests’ ability to detect infection will need to be evaluated with new variants, especially those with N-gene mutations (21, 22).

Given the short turnaround time, low cost, and ease of use, this test could play an important role in allowing children to limit absence from school and other activities while in quarantine or awaiting PCR test results, especially for asymptomatic children. It may assist in implementing test-to-stay strategies, where exposed school children are allowed to continue uninterrupted in-person learning given frequent testing for 1 week after exposure (23). The higher accuracy reported in our study also underscores the magnitude of missed opportunity in failing to make this important tool widely available to schools. If further studies with the extremely transmissible omicron variant show very high viral load and rapid antigen accuracy, these tests could become one of most valuable tools used to fight current and future COVID-19 variants.

## MATERIALS AND METHODS

### Study design.

This single-center prospective study compared the performance, quantified as sensitivity and specificity, of the BinaxNOW rapid antigen test against the current gold standard of RT-PCR. BinaxNOW is a lateral flow assay that targets the SARS-CoV-2 viral nucleocapsid (N) protein. The RT-PCR assay includes a panel of primer/probe sets targeting the viral N gene. This study was approved by the Johns Hopkins institutional review board.

### Participants and study site.

All individuals under 18 years old presenting for COVID-19 testing at the Baltimore Convention Center Field Hospital (BCCFH) COVID testing site were offered enrollment from 5/7/2021 to 12/6/2021. Potential enrollees and guardians were given written and verbal information about the study and given the opportunity to opt out of the additional anterior nares swabs for the rapid antigen test. The study site was a high-volume walk-up community collection site linked to the BCCFH.

### Data collection.

Before sample collection, data for demographics, symptoms, and any self-perceived exposure status were collected for each participant. Presence of active COVID-19 symptoms was assessed based on the standard Centers for Disease Control and Prevention (CDC) symptom checklist (24). Participants who reported at least one symptom were considered “symptomatic,” while those who reported no symptoms were considered “asymptomatic.” Participant exposure status was obtained according to CDC risk stratification. Participants were asked about living with someone with confirmed or suspected COVID-19; if they had been within 6 feet of someone with confirmed or suspected COVID-19 for more than 15 min; and if they had any other exposure to anyone with confirmed or suspected COVID-19. Participants were considered to have had high risk exposures if they lived with or were within 6 feet for more than 15 min of someone with confirmed or suspected COVID-19. Date of symptom onset and exposure were recorded. Test implementation, education, and training processes have been previously described (20).

### Sample collection.

The rapid antigen and RT-PCR samples were collected sequentially for each participant in the study by medical staff who were trained to perform rapid antigen tests. Manufacturer’s guidelines were followed in obtaining and processing rapid antigen samples (25). Staff collected bilateral anterior nares swabs first for direct inoculation onto rapid antigen tests followed by additional bilateral anterior nares samples for RT-PCR. Use of anterior nares samples for RT-PCR has been validated previously (26, 27). A designated, trained reader interpreted and recorded the result on site for each rapid antigen test 15 min after the test was administered per the instructions for use. All samples for RT-PCR were sent to the University of Maryland Pathology Associates—Maryland Genomics reference laboratory (University of Maryland School of Medicine, Baltimore, MD) and processed using a modification of the CDC 2019 Novel Coronavirus Real-Time RT-PCR Diagnostic Panel with off-line instrumentation under laboratory developed test (LDT) (Emergency Use Authorization application submitted and in pending status). It used N1 and N3 gene primers. Individuals for whom the RT-PCR or rapid antigen test was deemed indeterminate (control line not interpretable) were excluded from analysis. There was one inconclusive RT-PCR result, and no inconclusive rapid antigen results.

### Statistical analysis.

Accuracy of results (defined as sensitivity, specificity, and positive and negative predictive values [PPV and NPV, respectively]) and 95% confidence intervals (CIs) were calculated using the binomial exact method for the rapid antigen test for both symptomatic and asymptomatic populations compared with the RT-PCR gold standard. Accuracy results for symptomatic and asymptomatic groups were compared. Two-tailed *P* values were calculated using Fisher’s exact test. Similar test accuracy was calculated for exposure groups and age groups. Mean cycle threshold (C_T_) values for PCR-positive tests were compared for symptomatic and asymptomatic groups using independent sample T-test. A plot showing the distribution of rapid antigen C_T_ values was generated for symptomatic and asymptomatic individuals (Fig. 1). All statistical analyses were performed using JMP Pro, version 14.0.0, software (SAS Institute, Cary, NC).

## References

[1] Kates J, Tolbert J, Rouw A. 2021. An update on vaccine roll-out for 5–11 year-olds in the U.S. Kaiser Family Foundation, San Francisco, CA. https://www.kff.org/coronavirus-covid-19/issue-brief/an-update-on-vaccine-roll-out-for-5-11-year-olds-in-the-u-s/. Accessed 22 December 2021.

[2] American Academy of Pediatrics. 2021. Children and COVID-19 vaccination trends. http://www.aap.org/en/pages/2019-novel-coronavirus-covid-19-infections/children-and-covid-19-vaccination-trends/. Accessed 22 December 2021.

[3] Rosenberg ES. 2021. New COVID-19 cases and hospitalizations among adults, by vaccination status—New York, May 3–July 25, 2021. MMWR Morb Mortal Wkly Rep 70:1150–1155. doi:10.15585/mmwr.mm7034e1. 34437517 PMC8389393

[4] Li Ka Shing Faculty of Medicine. 2021. HKUMed finds Omicron SARS-CoV-2 can infect faster and better than Delta in human bronchus but with less severe infection in lung. The University of Hong Kong, Hong Kong, China. https://www.med.hku.hk/en/news/press/20211215-omicron-sars-cov-2-infection. Accessed 22 December 2021.

[5] Dorn E, Hancock B, Sarakatsannis J, Viruleg E. 2021. COVID-19 and education: the lingering effects of unfinished learning. McKinsey & Company, Atlanta, GA. https://www.mckinsey.com/industries/education/our-insights/covid-19-and-education-the-lingering-effects-of-unfinished-learning

[6] Chwe H, Quintana A, Lazer D, Baum M, Ognyanova K, Perlis R, Santillana M, Gitomer A, Green J, Druckman J, Simonson MD, Lin J. 2021. The COVID States Project #17: COVID-19 test result times. https://osf.io/rz34x/.

[7] Pekosz A, Parvu V, Li M, Andrews JC, Manabe YC, Kodsi S, Gary DS, Roger-Dalbert C, Leitch J, Cooper CK. 2021. Antigen-based testing but not real-time polymerase chain reaction correlates with severe acute respiratory syndrome coronavirus 2 viral culture. Clin Infect Dis 73:e2861–e2866. doi:10.1093/cid/ciaa1706. 33479756 PMC7929138

[8] Shaikh N, Friedlander EJ, Tate PJ, Liu H, Chang C-CH, Wells A, Hoberman A. 2021. Performance of a rapid SARS-CoV-2 antigen detection assay in symptomatic children. Pediatrics 148:e2021050832. doi:10.1542/peds.2021-050832. 34039718

[9] Sood N, Shetgiri R, Rodriguez A, Jimenez D, Treminino S, Daflos A, Simon P. 2021. Evaluation of the Abbott BinaxNOW rapid antigen test for SARS-CoV-2 infection in children: implications for screening in a school setting. PLoS One 16:e0249710. doi:10.1371/journal.pone.0249710. 33819311 PMC8021178

[10] CDC. 2020. COVID Data Tracker. Centers for Disease Control and Prevention, Atlanta, GA. https://covid.cdc.gov/covid-data-tracker. Accessed 1 September 2021.

[11] Maryland Department of Health. Coronavirus disease 2019 (COVID-19) outbreak. https://coronavirus.maryland.gov/. Accessed 15 March 2022.

[12] Food and Drug Administration. 2020. Policy for coronavirus disease-2019 tests during the public health emergency (revised): guidance for developers and food and drug administration staff. https://www.fda.gov/regulatory-information/search-fda-guidance-documents/policy-coronavirus-disease-2019-tests-during-public-health-emergency-revised. Template available at https://www.fda.gov/media/137907/download. Accessed on 14 June 2021.

[13] CDC. 2021. Transmission of SARS-CoV-2 in K-12 Schools. Centers for Disease Control and Prevention, Atlanta, GA. https://www.cdc.gov/coronavirus/2019-ncov/science/science-briefs/transmission_k_12_schools.html. Accessed 26 December 2021.

[14] Bolze A, Luo S, White S, Cirulli ET, Wyman D, Dei Rossi A, Machado H, Cassens T, Jacobs S, Barrett SMK, Tanudjaja F. 2021. SARS-CoV-2 variant Delta rapidly displaced variant Alpha in the United States and led to higher viral loads. Cell Rep Med 3:100564. 10.1016/j.xcrm.2022.100564 PMC8922438 35474739

[15] Li B, Deng A, Li K, Hu Y, Li Z, Xiong Q, Liu Z, Guo Q, Zou L, Zhang H, Zhang H, Ouyang F, Su J, Su W, Xu J, Lin H, Sun J, Peng J, Jiang H, Zhou P, Hu T, Luo M, Zhang Y, Zheng H, Xiao J, Liu T, Che R, Zeng H, Zheng Z, Huang Y, Yu J, Yi L, Wu J, Chen J, Zhong H, Deng X, Kang M, Pybus OG, Hall M, Lythgoe KA, Li Y, Yuan J, He J, Lu J. 2021. Viral infection and transmission in a large, well-traced outbreak caused by the SARS-CoV-2 Delta variant. medRxiv. doi:10.1101/2021.07.07.21260122. PMC8786931 35075154

[16] Pollock NR, Jacobs JR, Tran K, Cranston AE, Smith S, O’Kane CY, Roady TJ, Moran A, Scarry A, Carroll M, Volinsky L, Perez G, Patel P, Gabriel S, Lennon NJ, Madoff LC, Brown C, Smole SC. 2021. Performance and implementation evaluation of the Abbott BinaxNOW rapid antigen test in a high-throughput drive-through community testing site in Massachusetts. J Clin Microbiol 59:e00083-21. doi:10.1128/JCM.00083-21. 33622768 PMC8091851

[17] Prince-Guerra JL, Almendares O, Nolen LD, Gunn JKL, Dale AP, Buono SA, Deutsch-Feldman M, Suppiah S, Hao L, Zeng Y, Stevens VA, Knipe K, Pompey J, Atherstone C, Bui DP, Powell T, Tamin A, Harcourt JL, Shewmaker PL, Medrzycki M, Wong P, Jain S, Tejada-Strop A, Rogers S, Emery B, Wang H, Petway M, Bohannon C, Folster JM, MacNeil A, Salerno R, Kuhnert-Tallman W, Tate JE, Thornburg NJ, Kirking HL, Sheiban K, Kudrna J, Cullen T, Komatsu KK, Villanueva JM, Rose DA, Neatherlin JC, Anderson M, Rota PA, Honein MA, Bower WA. 2021. Evaluation of Abbott BinaxNOW rapid antigen test for SARS-CoV-2 Infection at two community-based testing sites – Pima County, Arizona, November 3–17, 2020. MMWR Morb Mortal Wkly Rep 70:100–105. doi:10.15585/mmwr.mm7003e3. 33476316 PMC7821766

[18] American Hospital Association. CDC ends indoor mask requirements for fully vaccinated people. https://www.aha.org/news/headline/2021-05-13-cdc-ends-indoor-mask-requirements-fully-vaccinated-people. Accessed 3 May 2022.

[19] Abbott. BinaxNOW COVID-19 Antigen Self Test: healthcare provider instructions for use. https://www.fda.gov/media/147254/download. Accessed 3 May 2022.

[20] Siddiqui ZK, Chaudhary M, Robinson ML, McCall AB, Peralta R, Esteve R, Callahan CW, Manabe YC, Campbell JD, Johnson JK, Elhabashy M, Kantsiper M, Ficke JR, CONQUER COVID Consortium. 2021. Implementation and accuracy of BinaxNOW rapid antigen COVID-19 test in asymptomatic and symptomatic populations in a high-volume self-referred testing site. Microbiol Spectr 9:e01008-21. doi:10.1128/Spectrum.01008-21. PMC8668078 34851137

[21] U.S. Food & Drug Administration. 2021. SARS-CoV-2 viral mutations: impact on COVID-19 tests. FDA, Silver Spring, MD. https://www.fda.gov/medical-devices/coronavirus-covid-19-and-medical-devices/sars-cov-2-viral-mutations-impact-covid-19-tests. Accessed 20 March 2022.

[22] Pu R, Liu S, Ren X, Shi D, Ba Y, Huo Y, Zhang W, Ma L, Liu Y, Yang Y, Cheng N. 2022. The screening value of RT-LAMP and RT-PCR in the diagnosis of COVID-19: systematic review and meta-analysis. J Virol Methods 300:114392. doi:10.1016/j.jviromet.2021.114392. 34856308 PMC8629515

[23] Nemoto N, Dhillon S, Fink S, et al. 2021. Evaluation of test to stay strategy on secondary and tertiary transmission of SARS-CoV-2 in K–12 Schools —Lake County, Illinois, August 9–October 29, 2021. MMWR Morb Mortal Wkly Rep 70:1778–1781. doi:10.15585/mmwr.mm705152e2. 34968375 PMC8736270

[24] CDC. 2021. Symptoms of COVID-19. Centers for Disease Control and Prevention, Atlanta, GA. https://www.cdc.gov/coronavirus/2019-ncov/symptoms-testing/symptoms.html. Accessed 22 December 2021.

[25] Abbott. 2020. BinaxNOW COVID-19 Ag card. https://www.fda.gov/media/141570/download. Accessed 16 August 2021.

[26] IDSA. 2020. Nucleic acid amplification testing (e.g. RT-PCR). https://www.idsociety.org/covid-19-real-time-learning-network/diagnostics/RT-pcr-testing/. Accessed 3 May 2022.

[27] Tu Y-P, Jennings R, Hart B, Cangelosi GA, Wood RC, Wehber K, Verma P, Vojta D, Berke EM. 2020. Swabs collected by patients or health care workers for SARS-CoV-2 testing. N Engl J Med 383:494–496. NEJMc2016321. doi:10.1056/NEJMc2016321. 32492294 PMC7289274

